# Detection of influenza C virus but not influenza D virus in Scottish respiratory samples

**DOI:** 10.1016/j.jcv.2015.11.036

**Published:** 2016-01

**Authors:** Donald B. Smith, Eleanor R. Gaunt, Paul Digard, Kate Templeton, Peter Simmonds

**Affiliations:** a Centre for Immunology, Infection and Evolution, University of Edinburgh, Ashworth Laboratories, Kings Buildings, West Mains Road, Edinburgh EH9 3JT, United Kingdom; b The Roslin Institute Building, The University of Edinburgh, Easter Bush Campus, Midlothian, EH25 9RG, United Kingdom; c Department of Virology, Royal Infirmary of Edinburgh, 51 Little France Crescent, Old Dalkeith Road, Edinburgh EH16 4SA, United Kingdom

**Keywords:** Influenza C virus, Respiratory disease

## Abstract

**Background:**

A newly proposed genus of influenza virus (influenza D) is associated with respiratory disease in pigs and cattle. The novel virus is most closely related to human influenza C virus and can infect ferrets but infection has not been reported in humans.

**Objectives:**

To ascertain if influenza D virus can be detected retrospectively in patient respiratory samples.

**Study design:**

3300 human respiratory samples from Edinburgh, Scotland, covering the period 2006–2008, were screened in pools of 10 by RT-PCR using primers capable of detecting both influenza C and D viruses.

**Results:**

Influenza D was not detected in any sample. Influenza C was present in 6 samples (0.2%), compared with frequencies of 3.3% and 0.9% for influenza A and B viruses from RT-PCR testing of respiratory samples over the same period. Influenza C virus was detected in samples from individuals <2 years or >45 years old, with cases occurring throughout the year. Phylogenetic analysis of nearly complete sequences of all seven segments revealed the presence of multiple, reassortant lineages.

**Conclusion:**

We were unable to detect viruses related to influenza D virus in human respiratory samples. Influenza C virus was less prevalent than influenza A and B viruses, was associated with mild disease in the young (<2 years) and old (>45 years) and comprised multiple, reassortant lineages. Inclusion of influenza C virus as part of a diagnostic testing panel for respiratory infections would be of limited additional value.

## Highlights

• “Influenza D” virus was not detected in Scottish respiratory samples (*n* = 3000).
• Influenza C virus infection was present in 0.2% of respiratory samples.
• Six influenza C virus complete genomes were sequenced.
• Influenza C isolates comprised multiple, reassortant lineages.

## 1 Objectives

The *Orthomyxoviridae* is a family of viruses with segmented negative sense RNA genomes that includes three genera of viruses associated with human disease: *Influenzavirus A (IAV)*, *Influenzavirus B* (IBV) and *Influenzavirus C* (ICV). IAV and IBV cause the majority of symptomatic influenza cases, have been studied intensively and are part of routine virological screening. ICV was discovered in a sample from an individual with respiratory illness [8], lacks a neuraminidase gene and consequently has a 7 segment genome compared to the 8 of IAV and IBV. Epidemiologic profiling of ICV consistently shows a relatively low frequency of infection, mostly in young or elderly individuals. The low prevalence of ICV and generally mild clinical outcome have discouraged the inclusion of ICV in routine virological screening.

A divergent influenza virus (prototype isolate C/Oklahoma/1334/2011, C/OK) associated with respiratory/influenza-like illness in pigs and cattle has recently been described [2], [3], [6], [7]. The low level of amino acid sequence identity (50%) between C/OK and ICV has led to the proposal (not yet ratified by the ICTV) that it represents a new genus, “*Influenzavirus D*” [7]; in this paper we refer to influenza C/OK and related viruses as influenza D virus (IDV). Since reactivity to IDV has been detected in human sera [6], and ICV can be transmitted between humans and pigs [9], it seemed possible that IDV might also infect humans.

## 2 Study design

To test the possibility that IDV is associated with human illness, we screened nucleic acid (produced by Biorobot MDx, Qiagen) from archived and anonymised respiratory samples collected in hospitals and primary care facilities in South East Scotland and deposited in a curated archive (NHS Lothian tissue bank, ethical approval 10/S1402/33) [4]. Retained epidemiologic data relating to the samples (ethical approval 08/S11/02/2) included patient age group, gender, referral source, collection month, any recorded clinical information, and the results of routine virological testing for adenovirus, IAV, IBV, parainfluenza viruses 1–3 (PIV1–3) and human respiratory syncytial virus (HRSV).

Degenerate primers were designed that would be capable of detecting the PB1 gene of ICV and porcine and bovine strains of IDV (Supplementary Table 1). PB1 was chosen since it is the least divergent (72% amino acid identity) between ICV and IDV sequences [6] with a single lineage amongst known IDV isolates [2]. Nucleic acids from individual samples obtained between August 2006 and June 2008 were pooled in groups of 10 and reverse transcribed using the A3500 reverse transcription system (Promega) and random primers, as part of a previous study [4]. cDNA, derived from 0.31 μl of each respiratory sample, was tested for the presence of IDV RNA by nested PCR in 20 μl reactions containing 5 μl cDNA, 4 μl 5×MgCl_2_ buffer, 0.2 μl dNTPs (3 μM), 0.1 μl GoTaq DNA polymerase (Promega), and 10 mM each outer primer for 35 cycles of 94 °C for 18 s, 50 °C for 21 s and 72 °C for 90 s, followed by 72 °C for 300 s. A second round of PCR was then performed with nested primers under the same conditions. Positive controls were a synthetic oligo of the IDV partial PB1 sequence (GeneArt by Invitrogen) and an ICV positive sample. Pools were screened twice, and if repeatedly positive, individual samples contributing to RT-PCR positive pools were identified by the same method. Amplicons were sequenced using BigDye (Life Technologies). Nucleotide sequences of individual genome segments from RT-PCR positive samples (Genbank accession numbers KT835335–KT835370) were assembled by sequence analysis of overlapping amplicons using nested pairs of primers specific for each segment (Supplementary Table 1).

## 3 Results

RT-PCR screening of 3300 respiratory samples resulted in the detection of 6 pools from which a single RT-PCR positive sample (0.2%) could be identified. A further seven pools were RT-PCR positive but a corresponding RT-PCR positive sample could not be identified. Sequence analysis revealed that all 13 amplicons were derived from ICV rather than IDV. IAV and IAB were detected by RT-PCR screening over the same period of 3.2% and 0.9% respectively.

In agreement with previous studies, cases of ICV infection occurred both in the summer and winter of 2007 (June, July, November, December ×3) in both the very young (<2 years, *n* = 4) and older individuals (>45 years, *n* = 2); all but one of the patients were hospitalised (Table 1). Symptoms were recorded only for the children and were diagnosed as bronchiolitis (*n* = 2), bronchitis (*n* = 1) and vomiting (*n* = 1). Coinfection with another respiratory virus was detected in three of the four children (PV-3, *n* = 1; HRSV, *n* = 2) and so it is not conclusive that ICV was the causative agent of illness in these cases. There was no evidence of clustering by residential postcode or place of admission.

**Fig. 1:**
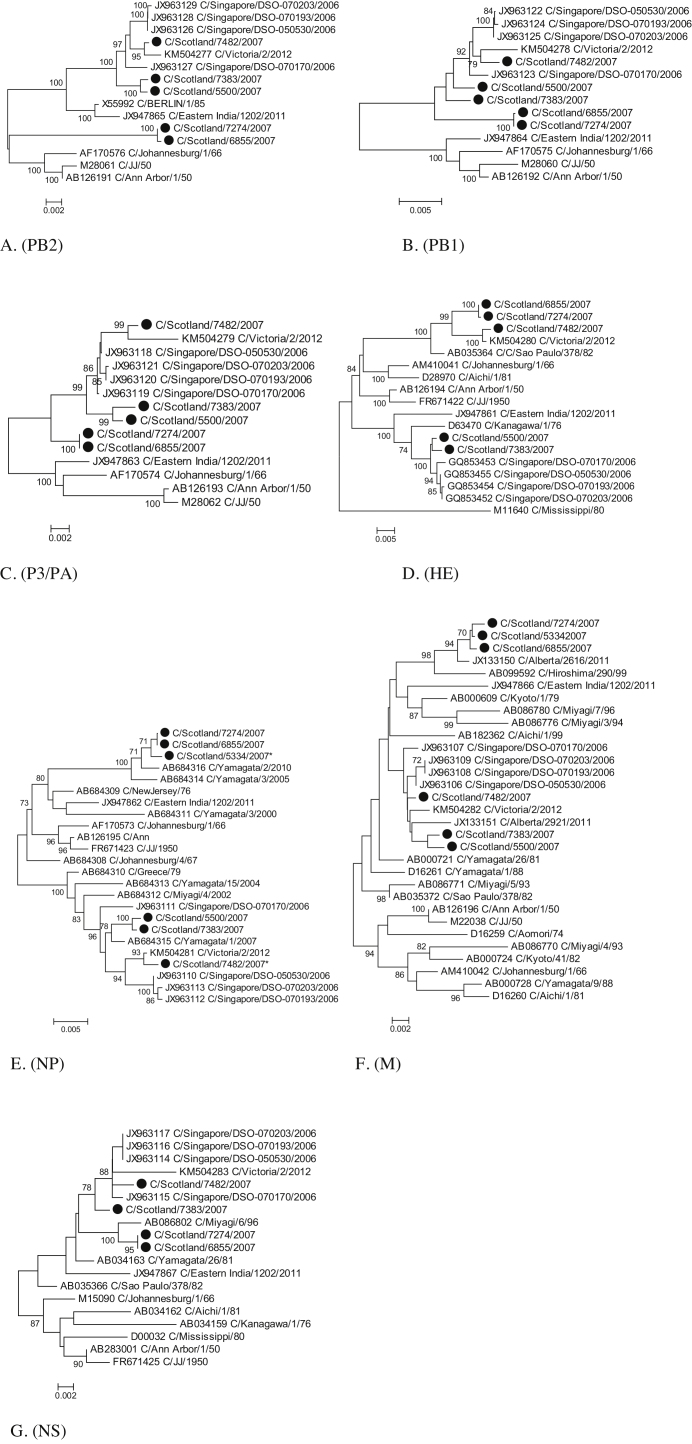
Phylogenetic analysis of individual influenza V virus genome segments. A. 1(PB2), B. 2 (PB1), C. 3 (PA/P3), D. 4 (HE), E. 5 (NP), F. 6 (M) and G. 7 (NS). For each segment, sequences included all completely sequenced isolates together with other isolates representing divergent or previously defined groupings and for which a nearly complete segment sequence was available. * indicates incomplete NP sequences. Neighbour joining trees were produced from maximum composite likelihood distances using MEGA 6 [17]. Numbers indicated branches supported by >70% of bootstrap replicates.

Complete genome sequences (>90% of all seven segments) have previously been obtained for only 9 ICV isolates, none of European origin. We sequenced some or all segments from the six Scottish ICV RT-PCR positive samples (Table 1, Fig. 1). Isolate 7482 grouped with C/Victoria/2/2012 in all seven segments, with the HE segment corresponding to C/Sao Paulo/378/82 lineage. Two isolates (5500 and 7383) only differed from this pattern in that the HE gene grouped with C/Singapore/DSO-070193/2006 (C/Kanagawa/1/76 lineage). Two isolates (6855 and 7274) had PB1, P3 and HE genes that grouped with C/Victoria/2/2012, but the other genes were phylogenetically distinct from previously reported complete genome sequences. Isolate 5334 was incompletely sequenced, but was closely related to isolates 6855 and 7274 in the NP and M genes.

## 4 Discussion

We were unable to detect viruses in humans related to IDV amongst our study group of respiratory samples from unwell or hospitalised individuals. Our degenerate primers were designed to match ICV and both porcine and bovine isolates of IDV [2] and so would be expected to detect divergent human variants of IDV. Although some human sera have haemagglutination-inhibition titres of 20 or more against IDV, this is much lower than the proportion with such titres against ICV (34%) [6]. This suggests that if viruses related to IDV do infect humans, they are relatively uncommon.

ICV was detected at a low frequency (0.2%) in both the summer and winter in patients <2 years or >45 years old, although the short period of our study (23 months) and previous evidence for periodic infection [16] makes generalisation difficult. However, the relatively low frequency of detection of ICV is similar to that observed in virus isolation studies of children with acute respiratory symptoms from Japan (0.2%, 0.38%, 0.5%) [11], [12], [13], and the Philippines (0.28%) [13], and in a molecular study of all age groups in Spain (0.7%) [1]. Somewhat higher frequencies of infection have been reported in a molecular study of IAV and IBV negative respiratory samples from Canada (2.58%) [14] and amongst Italian children with community acquired pneumonia (1.3%) [16]. Our failure to identify a RT-PCR positive sample in seven RT-PCR positive pools may reflect the degradation of RNA following prolonged storage; if these samples could have been identified the frequency of infection would have been 0.4%.

We confirmed previous reports of frequent reassortment between ICV lineages [10], [15] and the co-circulation of multiple lineages [1], [12], [13]. Discordant groupings were observed for C/East India 1202/2011 when comparing subgenomic or complete sequences of the PB1 and P3 genes consistent with recombination between lineages with breakpoints between nucleotide positions 413 and 443 of PB1, and 406 and 552 of P3. A study published prior to the isolation of this strain found no evidence for homologous recombination in ICV [5].

The low detection frequency of ICV in hospitalised Scottish patients, coupled with the high mixed infection frequency and lack of evidence for pathogenicity, suggests that routine diagnostic testing for this virus would be of limited value.

## Figures and Tables

**Table 1:** Clinical and virological features associated with ICV positive samples.

Isolates grouping with C/Victoria/2/2012 are indicated by filled boxes; black:strong grouping, grey:weaker grouping. KA76:C/Kanagawa/1/76 lineage, 6855:groups with C/Scotland/6855/2007, blank:not sequenced; PIV3:parainfluenza virus 3, HRSV:human respiratory syncytial virus.
