# Supplementary material for: Detection of influenza C virus but not influenza D virus in Scottish respiratory samples
Source: J Clin Virol. 2016 Jan;74:50–3. doi: 10.1016/j.jcv.2015.11.036 (PMC4710576; doi:10.1016/j.jcv.2015.11.036)
Supplement: Supplementary file 1 [file mmc1.docx]

Supplementary Table 1 Oligonucleotide primers

| Segment | region_sense_5’ base | Sequence (5’ ….-3’) |
| --- | --- | --- |
| PB1 (screening) | PB1_OS_939  PB1_OAS_1827  PB1_IS_1241  PB1_IAS_1718 | CWGGRGACAAYAGYAARTGGAATGA  TTGTTCATYARTTTCCCRCCATCWG  GGAGGAATGCTKATGGGAATGTT  AAGGRTGKACTCTRTAWGTYGCTC |
| PB1 | \| PB1_OS_16 \| \| --- \| \| PB1_IS_38 \| \| PB1_S_641 \| \| PB1_S_671 \| \| PB1_AS_721 \| \| PB1_AS_761 \| \| PB1_AS_1293 \| \| PB1_AS_1326 \| \| PB1_S_1636 \| \| PB1_S_1659 \| \| PB1_AS_2279 \| \| PB1_AS_2307 \| | GCAGRGGATTATGGAAATCAACC   \| CCATATTTGATGTTTCTAAACAACG \| \| --- \| \| GTACCTGTAATGGGAATTGACTC \| \| CATGATGAGTTTTTAATTAGAGC \| \| CATCTTTGGCCATGGTATTAATAG \| \| GTGTTGCAATTGCTCTTCTTTGC \| \| CTTACTCCAAGRACTGTTGACAGC \| \| GCTTTTAGTTCTTCATCCATATAAC \| \| TAAYAGCTTATCACCTTCTACTGC \| \| CTTTAATGGCCTTAAGGATATGTCTCC \| \| CAAKTCTAGCTTTCTTTGCATCC \| \| GGTTTAACAGCCCCTCCTCATCTAC \| |
| PB2 | \| PB2_S_24 \| \| --- \| \| PB2_S_51 \| \| PB2_S_752 \| \| PB2_S_779 \| \| PB2_AS_844 \| \| PB2_AS_870 \| \| PB2_S_1520 \| \| PB2_S_1558 \| \| PB2_AS_1609 \| \| PB2_AS_1636 \| \| PB2_AS_2296 \| \| PB2_AS_2358 \| | \| GTCTYTTCTATTGACAATAGCAAAGG \| \| --- \| \| ATACAAAARACTATGCCAAGATGC \| \| GAGGAGAAACTTGGATACAAGAAGC \| \| CTGCAGGRATTTCCAATGTTGATC \| \| GACAAACTTTYCTACATACTGCTC \| \| GACAAACTTTYCTACATACTGCTC \| \| CAGGAAAGATTGAYATGCCAGAATC \| \| CACCTTGAGCTCTCTGATAATCTTG \| \| CAATTCTTCCTGATGAATCAAATGC \| \| CACTAGGAAGRTCTAAAATTGTTGC \| \| CATTCTTATTTACATCGCTTGATGC \| \| GCAAGAGGATTTTTAGTTAGACATC \| |
| P3 | \| P3_S_29 \| \| --- \| \| P3_S_61 \| \| P3_S_750 \| \| P3_S_781 \| \| P3_AS_839 \| \| P3_AS_879 \| \| P3_S_1383 \| \| P3_S_1420 \| \| P3_AS_1477 \| \| P3_AS_1509 \| \| P3_AS_2105 \| \| P3_AS_2133 \| | \| AAACTTTTGCCGAAATAGCAGAG \| \| --- \| \| GAGCCAGAAGCTGTAAGAATAGC \| \| GAATAAATTTCGACAAATACGTCTACC \| \| GGACCAATGGCACCCTATACCC \| \| TTTGTAAACATCCATGCTTCTTCC \| \| GAGAATTTCACCAGCTCTTGATC \| \| GAAAATAAAAGTGGTGCCCATATATGC \| \| GAAAGGAAATCAATGCAAGAAGG \| \| CCAAATAAACAGTCCATTTCTGAGG \| \| GTTTAAATGTGATTTTGAYTTGACGC \| \| ACAAGTTCATTCAACCTTTGAGCC \| \| CTTAGCGCCTCTTTTTGAGGCCTC \| |
| HE | \| HE_S_108 \| \| --- \| \| HE_S_134 \| \| HE_S_740 \| \| HE_S_771 \| \| HE_AS_826 \| \| HE_AS_850 \| \| HE_S_1299 \| \| HE_S_1357 \| \| HE_AS_1411 \| \| HE_AS_1451 \| \| HE_AS_1996 \| \| HE_AS_2021 \| | \| GATATGCCTTCAAAAGCAAGTG \| \| --- \| \| GAAYAGTAGCTTCAGCCTACACAATGG \| \| GCARAMTGCATCTTGTGGCTTCTTGC \| \| YATYTATGAYAGYAAAGAAGTG \| \| CTTGAAAGTAGTTGYCACATCCTG \| \| CYTTTCCAGATGAATCRTAGATCAC \| \| GGCTGCAAARGAAGAATCHAYTCC \| \| GGAACYGATACCACTGTAACYAAACC \| \| GGTCATCRATKCCAAAAATYCTGC \| \| CCTGYTTCAACGATTGCAACAAAG \| \| CAGAGATCACCAAAGCTGCCAATG \| \| CATTTAGTTCKGCAGATGGCGATCC \| |
| NP | \| NP_OS_32 \| \| --- \| \| NP_IS_50 \| \| NP_Int_OS_864 \| \| NP_Int_IS_886 \| \| NP_Int_IAS_980 \| \| NP_Int_OAS_1009 \| \| NP_IAS_1729 \| \| NP_OAS_1744 \| | \| GTCTGACAGACGTCAAAACAG \| \| --- \| \| CAGAAAGACGCCAGATGAGCA \| \| ATGACAATGCCTTGGTGTATG \| \| GGTTGGCTGCCAGACTTACTC \| \| CTCCATTGCAATATTGAAGAC \| \| GTCCCTTGAAAGGAACAAATTCC \| \| CTTTAATTTTYTCCAGGAATGTTG \| \| ATGYGATGAATTTAATCTGAC \| |
| M | \| M_OS_27 \| \| --- \| \| M_IS_59   \| M_S_420 \| \| --- \| \| M_S_449 \| \| M_AS_502 \| \| M_AS_545 \| \| \| M_IAS_1088 \| \| M_OAS_1118 \| | \| TGGCACATGAAATAYTRATTGCYG \| \| --- \| \| GCATTTCTAAAAAATGTTGCTCCTG   \| CAGACGAYTAYACACCRAGACATCCG \| \| --- \| \| GGRACAATYACAGCYTGGTTRAGATG \| \| CTYCCACYTTCTGAGAYATTRCTC \| \| TTGCTGTGCTKGCTTTTCTYACTTC \| \| \| CATCCTCTCCCAGRTCAAGTCTC \| \| GAATTGGTGAGTTGTCGGTTTCGTC \| |
| NS | \| NS_OS_40 \| \| --- \| \| NS_OS_69 \| \| NS_IAS_871 \| \| NS_OAS_890 \| | \| CAGTCAAATCAACAAATTTAATGGC \| \| --- \| \| RTAGCCACAAAAATGTTAGAGAG \| \| CACARAGATTTTATCATYAATATARTG \| \| CAATTATATAAGTGAAKTRCACARAG \| |
